# ACE2 diversity in placental mammals reveals the evolutionary strategy of SARS-CoV-2

**DOI:** 10.1590/1678-4685-GMB-2020-0104

**Published:** 2020-06-08

**Authors:** Bibiana S.O. Fam, Pedro Vargas-Pinilla, Carlos Eduardo G. Amorim, Vinicius A. Sortica, Maria Cátira Bortolini

**Affiliations:** 1 Universidade Federal do Rio Grande do Sul, Instituto de Biociências, Departamento de Genética, Porto Alegre, RS, Brazil.; 2 Universidade de São Paulo, Faculdade de Medicina de Ribeirão Preto, Departamento de Bioquímica e Imunologia, Ribeirão Preto, SP, Brazil.; 3 University of Lausanne, Departament of Computational Biology, Lausanne, Switzerland.

**Keywords:** ACE2, placental mammals, SARS-CoV-2, COVID-19, inter and intra-species diversity

## Abstract

The recent emergence of SARS-CoV-2 is responsible for the current pandemic of COVID-19, which uses the human membrane protein ACE2 as a gateway to host-cell infection. We performed a comparative genomic analysis of 70 ACE2 placental mammal orthologues to identify variations and contribute to the understanding of evolutionary dynamics behind this successful adaptation to infect humans. Our results reveal that 4% of the ACE2 sites are under positive selection, all located in the catalytic domain, suggesting possibly taxon-specific adaptations related to the ACE2 function, such as cardiovascular physiology. Considering all variable sites, we selected 30 of them located at the critical ACE2 binding sites to the SARS-CoV-like viruses for analysis in more detail. Our results reveal a relatively high diversity of ACE2 between placental mammal species, while showing no polymorphism within human populations, at least considering the 30 inter-species variable sites. A perfect scenario for natural selection favored this opportunistic new coronavirus in its trajectory of infecting humans. We suggest that SARS-CoV-2 became a specialist coronavirus for human hosts. Differences in the rate of infection and mortality could be related to the innate immune responses, other unknown genetic factors, as well as non-biological factors.

## Introduction

The novel coronavirus SARS-CoV-2 (denominated before as 2019-nCOV) is a single-stranded RNA virus member of the Coronaviridae family. The estimates of the most recent common ancestor (MRCA) for the four coronavirus genera of this family range from 10,000 to millions of years (Wertheim *et al.*, 2013). The most commented member of the Coronaviridae family at the moment, SARS-CoV-2, has a very recent origin. Based on genome sequence from different SARS-CoV-2 strains and a yearly mutation rate of 1.24 × 10^−3^ per site, Li *et al.* (2020a) estimated that it originated on November 24 2019.

SARS-CoV-2 is responsible for the current pandemic of Corona Virus Disease 2019 (COVID-19) (Chan *et al.*, 2020; Coronaviridae Study Group, 2020; Huang *et al.*, 2020; Wang *et al.*, 2020). The COVID-19 was first reported in Wuhan (China) in December 2019 and spread worldwide, with a high rate of transmission, thousands of infected patients and many deaths between December 2019 and April 2020, and these numbers continue to grow. On March 11, World Health Organization (WHO) characterized COVID-19 as a pandemic, the first one caused by a coronavirus (WHO, situation report, March 11, 2020). SARS-CoV-2 causes a severe respiratory syndrome in humans, being transmitted arguably through different routes (*i.e*., fomites, airborne or fecal-oral) by an animal to human and human to humans (Chan *et al.*, 2020; Huang *et al.*, 2020; Li R *et al.*, 2020), similar to SARS-CoV (Severe Acute Respiratory Syndrome-related coronavirus) and MERS-CoV (Middle East Respiratory Syndrome-related coronavirus). SARS-CoV was initially transmitted to humans by the palm civet (*Paguma larvata*; Kan *et al.*, 2005) and MERS-CoV by the dromedary (*Camelus dromedarius*; Zaki *et al.*, 2012). SARS-CoV-2 is classified as *Beta coronavirus* subgenus *Sar becovirus* with ∼70% of similarity with SARS-CoV (Wu *et al.*, 2020). Genetic studies from different research groups found that SARS-CoV-2 is highly similar to the bat (*Rhinolophs affinis*) coronavirus (Guo *et al.*, 2020; Ji *et al.*, 2020; Li R *et al.*, 2020; Wu *et al.*, 2020; Zhou *et al.*, 2020). Other studies comparing genomes of different coronaviruses showed local genomic similarities between the human coronavirus and those from snakes, turtles, pangolins, and minks, suggesting these animals as possible intermediate-hosts (Guo *et al.*, 2020; Ji *et al.*, 2020; Li X *et al.*, 2020; Liu *et al.*, 2020; Wan *et al.*, 2020; Wu *et al.*, 2020). The most recent metagenomic study on the topic reinforces the idea that pangolins (*Manis javanica*) should be considered as the most plausible intermediate-host of SARS-CoV-2 related viruses, and that they acquired these viruses independently from bats or another animal hosts (Lam *et al.*, 2020). The authors found putative recombination signals between the pangolins coronaviruses, bat coronaviruses, and human SARS-CoV-2 (Lam *et al.*, 2020), which highlights the complexity of the origin of the new human coronavirus. Although there are uncertainties about the original and intermediate hosts, mutation and/or recombination among coronaviruses may have enabled cross-species infection (Letko *et al.*, 2020; Wan *et al.*, 2020).

Similar to SARS-CoV, SARS-CoV-2 utilizes the Angiotensin-Converting Enzyme 2 (ACE2) as target site to infect cells (Li *et al.*, 2003; Li *et al.*, 2005a,b; Chen *et al.*, 2020; Hoffmann *et al.*, 2020; Letko *et al.*, 2020; Li F *et al.*, 2020; Li R *et al.*, 2020; Luan *et al.*, 2020; Walls *et al.*, 2020; Wan *et al.*, 2020; Xu *et al.*, 2020). *ACE2* gene is located on chromosome X, and its product ACE2, a zinc metallopeptidase protein, comprises 805 amino acids containing a single catalytic domain. ACE2 is mainly expressed at vascular endothelium, myocardium, lungs, kidneys, and intestines (Turner *et al.*, 2004; McKinney *et al.*, 2014; Zhang *et al.*, 2020). It is a strict carboxypeptidase that hydrolyzes its substrate removing a single amino acid from their respective C-terminal. It is responsible for cleaving Angiotensin I and II into peptides Angiotensin 1-9 and Angiotensin 1-7, respectively, both are key elements connected with cardiovascular physiology, regulation of vascular tonus, blood pressure, electrolyte balance, and water intake (Millan *et al.*, 1990; Donoghue *et al.*, 2000; Oudit *et al.*, 2009; McKinney *et al.*, 2014).

ACE2 is a cell-surface non-raft protein with little intracellular localization. When binding to coronaviruses, the protein internalizes down-regulating activity from the cell surface (Hamming *et al.*, 2007). After SARS-CoV-like viruses and ACE2 attach, molecular cascade signal events result in entry of the coronavirus into the host cell (Simmons *et al.*, 2013), showing a successful evolutionary infection strategy. Kuba *et al.* (2005) showed that ACE2 down regulation mediated by SARS-CoV contributes to the acute lung injury, the most important clinical implication of human respiratory diseases caused by a coronavirus.

The success of a zoonotic spillover (transmission of a pathogen from a vertebrate animal to a human) is a complex process. It depends on several cultural, ecological, and climatic conditions, as well as features of viruses and hosts (Olival *et al.*, 2017). The efficiency in recognizing the host binding receptor is a crucial step, which defines the preference of the virus for a given species, tissue or cell type. The interaction of SARS-CoV-2 spike (S) glycoprotein, through its receptor-binding domain (RBD), happens via an optimized binding to human ACE2 domains (Andersen *et al.*, 2020; Hoffman *et al.*, 2020; Walls *et al.*, 2020). According to recent studies, five amino acid changes associated with natural selection in the critical binding sites (L455, F486, Q493, S494, N501) located at the SARS-CoV-2 S glycoprotein would be responsible for this high tropism with human ACE2 (Andersen *et al.*, 2020; Wan *et al.*, 2020). Furthermore, S glycoprotein of SARS-CoV-2 contains a cleavage site for furin proteases at the junction of subunits S1 and S2 (Coutard *et al.*, 2020). During viral infection, S glycoprotein needs to be cleaved by host-cell proteases into S1 (which contains RBD) and S2 subunits. After this cleavage, the exposition of the S2 mediates fusion of the viral and host-cell membranes (Yan *et al.*, 2020). Furin proteases are abundant in the respiratory tract, and this characteristic of the SARS-CoV-2 has been considered to be essential for successful infection of SARS-CoV-2 in human cells (Lam *et al.*, 2020). In contrast, the highly related bat and pangolin SARS-CoV-2-like coronavirus do not have the furin cleavage site, which in principle could be a barrier to zoonotic coronavirus spillover. Moreover, human-infecting coronaviruses such as HCoV-OC43, MERS-CoV, and HKU1 also have been demonstrated to be cleaved at an S1/S2 cleavage site by furin proteases (Coutard *et al.*, 2020). However, in SARS-CoV, furin-mediated cleavage of the S glycoprotein appears not to occur naturally (Simmons *et al.*, 2004) and the introduction of a functional furin cleavage site in the S1/S2 junction of SARS-CoV S glycoprotein resulted in a dramatic enhancement of cell-cell fusion (Follis and Nunberg, 2004).

Changes in key amino acids (AA) related to an efficient interaction between SARS-CoV-like S glycoprotein and ACE2 are crucial to cross-species infection, multi-host infections, as well as differences in susceptibility to disease and its symptoms in animal species. The evolutionary phenomenon can be evoked to explain at least part of these events and conditions. To contribute to a better understanding of this complex process, we performed a comparative genomic analysis of 70 ACE2 orthologues, representative of placental mammal species, including pangolin, civet, bat, mink, and five New World monkey (NWm) species (Parvorder Platyrrhini). Furthermore, we describe the genetic variation of key binding sites across human populations. Our immediate goal is to reveal the taxon-specific variability in ACE2 amino acid sequence, its possible influence in cross-species SARS-CoV-2 infection, potential hosts, and other related topics that can be important and very timely considering the world outbreak of COVID-19.

## Material and Methods

### Sequences

Coding sequences for *ACE2* gene for 70 placental mammals species were retrieved trough BLAST from GenBank (www.ncbi.nlm.nih.gov accessed at 20/02/2020). For these analyses we considered 16 domestic and 54 wild species including representatives of orders Carnivora (N = 9), Ungulata (N = 11), Chiroptera (N = 10), Primates (N = 22), Rodentia (N = 9), and others (Table S1). Sequences were aligned according to the MUSCLE algorithm (Edgar, 2004) in AliView software (Larsson, 2014).

To investigate the variation within *Homo sapiens*, we used the Ensembl database (www.ensembl.org; Hunt. *et al.*, 2018) and the UNIPROT database (www.uniprot.org, The Uniprot Consortium, 2019). Both platforms used data from the 1000 Genome Project.

### Evolutionary Analysis

The consensus phylogeny of species, based on neutral molecular markers, was obtained from the Timetree server (www.timetree.org; Kumar *et al.*, 2017). This phylogeny is used as a reference by specialized literature following Meredith *et al.* (2011). To better understand evolutionary patterns that are acting on *ACE2,* we performed a Site Model (NsSite) test with Codeml package in PAML 4.9 software (Yang, 2007 *),* which allows for inter-specific phylogenetic comparison of substitution rates in protein-coding genes. This test allows for ω variation (ω = dN/dS, being dN the rate of non- synonymous substitutions and dS the rate of synonymous substitutions) across sites. It fits neutral (ω≅1), selective constraint (ω<1), or positive selection (ω>1) models to the observed levels of variation. To determine which evolutionary model best fits the analyzed data, we performed Likelihood Ratio Tests (Yang, 1998). For these, we compared the neutral model (M1) against the model that allows positive selection (M2a) with df = 2 (Yang, 2007). Bayes Empirical Bayes (BEB) was used to infer sites with a high probability of being under positive selection (Yang *et al.*, 2005). Furthermore, we classify amino acid variation found in placental mammals *ACE2* coding sequences into four classes of chemical similarity accordingly Grantham score (Grantham, 1974): conservative (GS = ≤50), moderately conservative (GS = 51–100), moderately radical (GS = 101–150), and radical (≥151).

## Results

**Figure 1:**
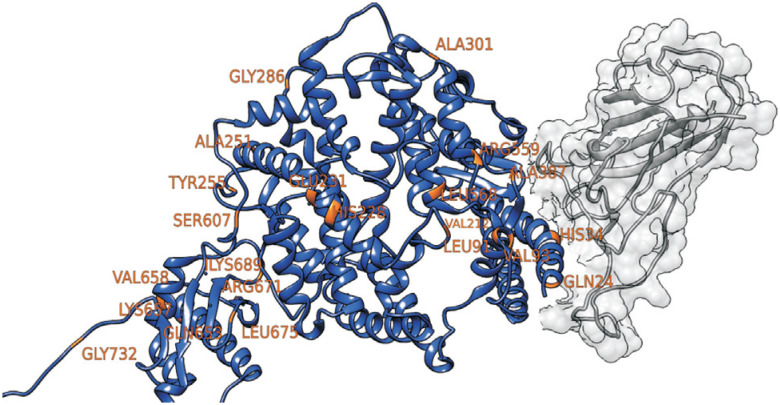
Structural analysis of sites under positive selection in placental mammals (orange) of human ACE2 (blue) binding to SARS-CoV2 spike glycoprotein (gray). The Protein Data Bank (PDB) ID is 6M17 (Yan *et al.*, 2020).

Our results reveal that the M2 model (NsSite; ω > 1) fits the placental mammal *ACE2* data significantly better than the neutral model (ω2 = 2.58680, *p* < 0.001; Table S2). Considering the M2 model, 4% of the *ACE2* sites are under positive selection, a value high enough to infer a non-neutral molecular evolution for this gene as whole, at least considering placental mammals. Note however, that this evolutionary scenario is not homogeneous across the different domains of the ACE2 protein. A parallel analysis, Bayes Empirical Bayes (BEB), highlights 22 sites (24, 34, 91, 93, 212, 228, 231, 251, 255, 286, 301, 387, 559, 568, 607, 653, 657, 658, 671, 675, 689, and 732) (Table S3; Figure 1) with a high probability (> 95%) of being under positive selection, all of them being in the active sites at the extracellular portion of the protein (residues 18-740), where the ACE2 catalytic domain is located. Of the remaining ACE2 sites, 69% are under functional constraint due to the negative selection (ω0 = 0.10125), while 27% are with selective relaxation (ω1 = 1). For instance, the signal peptide (1-17), transmembrane domain (741-761), and cytoplasmatic domain (761-805; see ω3 column of the Table S4) are more conserved, showing little variation across species, none of them with positive selection signals (Tables S2 and S4). We note that ACE2 is a critical element in the cascade of regulation of blood pressure and cardiovascular condition, among other functions, and that these AA changes with a significant signal of positive selection could be taxon-specific, related to particular adaptive phenotypes found in some of these diverse placental mammals.

**Figure 2:**
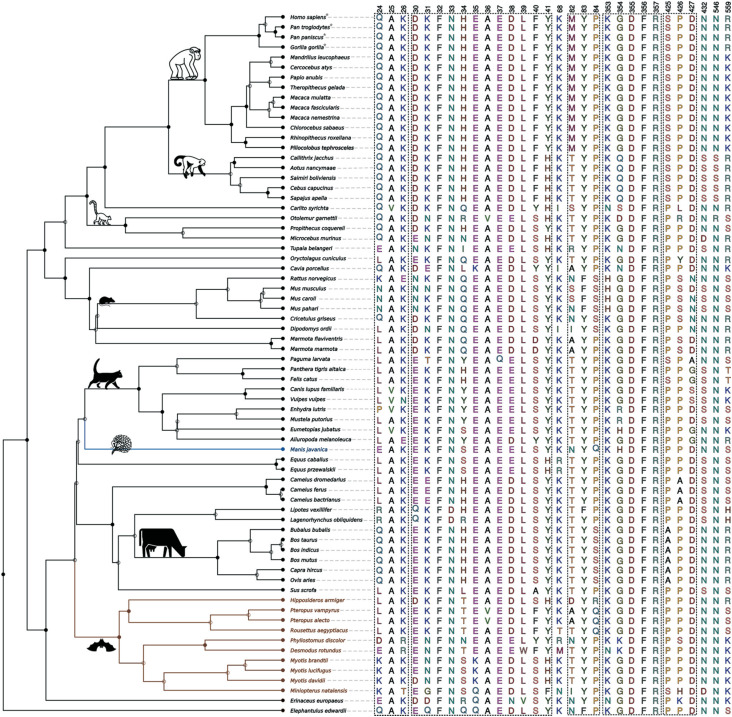
Crucial variable sites in placental mammalian ACE2 protein in the interaction with SARS-CoV-2. In color branches potential sources of SARS-CoV-2; in blue, Pangolin (*Manis javanica*) branch, and in brown bat (Chiroptera) branches.* Species whose susceptibility to infection by SARS-CoV-2 may be similar to human. The full nodes in the tree are those in which we have a taxonomic classification defined as order, family, gender, etc., while those with an empty node, this information is not well supported. Animal icons available in Noun project (https://thenounproject.com/).

After that first evolutionary analysis, we examined all variable sites found considering the 70 orthologues. However, we choose only the 30 variable sites with information that they are functional and/or involved in the interaction between host-ACE2 and SARS-CoV-like viruses (Towler *et al.*, 2004; Li F *et al.*, 2005; Li R *et al.*, 2020; Luan *et al.*, 2020; Wan *et al.*, 2020). Figure 2 shows the AAs observed in 30 chosen variable sites across the ACE2 orthologues of 70 selected placental mammal species. Three of them (24, 34, and 559) are target sites for positive selection, according to BEB (Table S3). The topology of Figure 2 recovers the phylogenetic relationship between the major taxonomic groups investigated. For example, *Homo sapiens* and all other great apes (family Hominoidea) share the same amino acids in the 30 positions, and only one divergence from Cercopithecidae species at the positive selection site 559 (conservative AA change, R559K; GS = 26) (Figure 2).

Noteworthy, our purpose here is not to report each variation observed in the 70 orthologues (Figure 2), but to point out some thought-provoking findings to support the discussion and the main conclusion of the present study. For this, we built Figure S1, a simplified version of the data from Figure 2, where only sites and species commented in the next paragraphs are shown.

Position 24, which is one of the three sites with high probability of being under positive selection, has eight different amino acids segregating across taxon (Figures 2 and S1). Li W *et al.* (2005) showed that the amino acid change from Glutamine to Lysine at position 24 (Q24K), together with a change from Lysine to Glutamate at position 26 (K26E), slightly inhibits interactions with SARS-CoV S glycoprotein (Li W *et al.*, 2005). *Rattus norvegicus* has this combination (24K and 26E), while mouse species (*Mus musculus, Mus caroli,* and *Mus pahari*) have an Asparagine (N) at the position 24. *Rattus norvegicus* ACE2 also has the combination of Asparagine (N), Phenylalanine (F), and Serine (S) at positions 82-84. According to Li W *et al.* (2005), this combination in rats (*R. norvegicus*) inhibits the interaction between ACE2 and SARS-CoV S glycoprotein. In addition, a Histidine (H) at positions 353 of ACE2 in the murine species also could inhibit or reduce the efficiency of the interaction according to Wan *et al.* (2020). Our study shows that murine species’ ACE2 have only 56% of identity with the human ACE2 regarding these 30 binding sites. This reinforces the idea that they cannot naturally bind to S glycoprotein of SARS-CoV-2. On the other hand, Glutamine (Q) and Lysine (K) at 24 and 26 ACE2 positions are found in humans and all primates, as well as in *Cavia porcellus* (Guinea-pig), a rodent used very often as an experimental animal. However, there are differences in other important binding sites, resulting in the identity between Guinea-pig ACE2 and the human ACE2, regarding these 30 binding sites, being just 70%.

Cat (*Felis catus*) and dog (*Canis lupus familiars*) are of special interest due to their close relationship with humans. Their ACE2 orthologues present at 77% and 73% of identity with the human ACE2 respectively, considering the 30 variable sites. Both species present identity with other species belonging to the Carnivora order, such as Leucine (L) at position 24. However, they also show differences from each other at positions 25, 34, 427, and 559, two of them are a target for positive selection (34 and 559; Table S3). In other words, 50% of the differences between the ACE2 binding sites of cat and dog are taxon-specific variants that may be connected with specific functions of the ACE2 protein in each of these two domesticated species.

Interestingly, the five NWm and Prossimian species show different patterns than other primates at position 82-84. They present a moderately conserved change at position 82 (M82T; GS = 81), which also represents a change in polarity. We note that, due to the great number of species that present the amino acid Threonine in that position, according to the principle of parsimony, Threonine is probably the ancestral allele, meaning that a non-synonymous mutation promoted the AA alteration Threonine (T) > Methionine (M) in the ancestral branches of the Catarrhini clade. However, the ancestor of the Platyrrhini maintained the ancestral allele T. At position 41, we also found the same amino acid in NWm and Prossimian species. The moderately conservative modification Y41H (GS = 83) represents changes in charge, once Histidine (H) is an acid AA. Another mutation at this same site (Y41A; GS = 112) implies a moderately radical change and polarity difference, since Tyrosine (Y) is a polar AA, while Alanine (A) is a non-polar AA. This type of alteration could potentially prevent viral interaction, at least with SARS-CoV according Li *et al.* (2005b). Based on the distribution of the AAs at position 41 of ACE2, it seems that both NWm and Prossimian clades shared the derived allele (H), also seen in the bat genera *Hipposideros* and *Myotis*, as well as in *Equus*, suggesting molecular convergence.

NWm ACE2 is also different from other Primates species due to two changes in the potential glycosylation sites at positions 432 and 546 (Luan *et al.*, 2020), where Arginine (N) was replaced by Serine (S). The glycosylation was suggested by the presence of electron density at all Arginine-linked site of the ACE2 N-terminal domain, including 432 and 546 and the loss of glycosylation sites may have functional implications (Towler *et al.*, 2004). Studies with MERS-CoV and with its host-cell entry receptor, DPP4, indicated that mutations that knock out glycosylation sites present in mouse DPP4 blocked the binding with MERS-CoV RBD (Peck *et al.*, 2017). The last authors concluded that glycosylation in DPP4 orthologue sites are a substantial barrier to MERS-CoV infection, particularly when combined with taxon-specific changes (Peck *et al.*, 2017).

We also observed a Serine (S) at the positively selected site 559, which reduces or inhibits the interaction of SARS-CoV S glycoprotein with ACE2 (Li W *et al.*, 2005). A Serine in that position is found not only in rodents but also in some species of carnivores that were already considered as possible original or intermediate hosts, such as the pangolin (*Manis javanica*). Pangolin ACE2 has ∼85% of similarity with human ACE2 (Lam *et al.*, 2020), but only 66% considering just these 30 sites investigated here. In addition, a Serine at position 559 is also observed in the two species of the Mustelidae family, ferret (*Mustela putorius*) and sea otter (*Enhydra lutris*).

Finally, we also leveraged intra-specific variation of the *ACE2* gene using data from human populations available in the 1000 Genomes Project (accessed through Ensembl and UNIPROT platforms). This dataset revealed 660 missense variants (all with minor allele frequency of 0.005 or less), 260 synonymous variants, 14,352 intron variants, 67 5’UTR variants, and 268 3’UTR variants in the *ACE2* gene in humans. Many of these have been investigated in case-control or genome-wide association studies for cardiovascular diseases and other correlated conditions (see for instance Ji *et al.*, 2017). Regarding the 30 ACE2 binding sites selected to present study (Figure 2), only one of them, at position 26, has two alleles segregating (K and R; Minor Allele Frequency or MAF = 0.002; rs4646116). Although we do not have population data for the other species investigated here, we observed that there is no relevant polymorphism regarding the 30 sites that promote the interaction between host-ACE2 and SARS-CoV-like viruses in the most studied species of all (*Homo sapiens*). This finding reinforces the idea that the AAs presented in Figure 2 are taxon-specific, *i.e*., characteristic of a species or a taxonomic group, where intra-specific variation is absent or negligible. Also, the AA conservation within *Homo sapiens* in these 30 sites indicates an instigating evolutionary constraint whose reasons need to be further studied. A search in the genome of the Neanderthal (*Homo neanderthalensis*) and in the specimen of Denisova indicates that both present the same AAs as *Homo sapiens* in these 30 sites.

## Discussion

Species of the Coronaviridae family can infect a wide variety of animals and humans, causing severe respiratory, enteric, hepatic, and neurological diseases (Weiss and Leibowitz, 2011). In humans, coronavirus has caused mainly respiratory tract infections and, for decades, were considered with little clinical and epidemiological importance until SARS-CoV and MERS-CoV outbreaks (Kuiken *et al.*, 2003; Zaki *et al.*, 2012). Today, SARS-Cov-2 is responsible for the COVID-19, a pandemic that has challenged governments and peoples of every country in the world. COVID-19 already killed more persons than diseases caused by SARS-CoV and MERS-CoV combined, emphasizing the importance of this pathology as a highly relevant public health concern (WHO; https://www.who.int/emergencies/diseases/novel-coronavirus-2019).

SARS-CoV-like viruses use ACE2 as their gateway to the host cell. Figure 2 illustrates the diversity of 70 ACE2 orthologues, considering 30 potential binding sites to the S glycoproteins of the SARS-CoV-like viruses. This fact opens up a wide range of possibilities for this type of coronavirus, whose rate of evolution is high, due to its high rates of mutation and recombination (Li *et al.*, 2020a). These phenomena that generate variability in the RNA viruses explain why they change host more frequently and jump between distantly related species more often than other pathogens (Woolhouse *et al.*, 2005a,b; Davies and Pedersen, 2008; Longdon *et al.*, 2014). These facts would explain a predicted origin of SARS-CoV-2 from original and/or intermediate phylogenetically distant hosts (for instance, bat and pangolin; Guo *et al.*, 2020; Ji *et al.*, 2020; Lam *et al.*, 2020; Li X *et al.*, 2020; Wu *et al.*, 2020; Zhou *et al.*, 2020).

It was also known that specific pathogen mutations are often required to enhance pathogen's fitness in a new host (Longdon *et al.*, 2014). This is exactly what happened for the emergence of SARS-CoV-2. Recent investigations suggest the jump across species that resulted in the emergence of SARS-CoV-2 in humans was mainly due to five amino acid changes in critical S glycoprotein binding sites (Wan *et al.*, 2020; Andersen *et al.*, 2020). In addition to that, the S glycoprotein of SARS-CoV-2 contains a cleavage site for furin proteases at the junction of subunits S1 and S2 as other human coronavirus (HCoV-OC43, MERS-CoV, and HKU1), but differently than what occurs with bat and pangolin SARS-CoV-2-like coronavirus, and SARS-CoV (Coutard *et al.*, 2020). These events are associated with the high and efficient tropism with human ACE2 (Andersen *et al.*, 2020; Lam *et al.*, 2020).

As opposed to the key differences observed across the 70 orthologues in mammals, no relevant polymorphism was identified in these 30 ACE2 binding sites considering human populations. In that sense, *Homo sapiens* is a “homogeneous” primate species, at least in regards to the 30 ACE2 important binding sites. In addition to that, *Homo sapiens* has a large population (census size of ∼7.5 billion individuals) currently living in virtually all habitable places on Earth, including regions with very high demographic density. Based on these observations, we can suggest that the high and efficient tropism is extended to all human populations, in addition to demographic and cultural conditions linked to particular human populations that may facilitate contagion and dispersion of this opportunistic coronavirus.

Hosts and pathogens are always in an evolutionary arms race. Several investigations reveal that coronavirus evolved to recognize different host-receptors, while others explain the host's strategies to defend themselves. For example, Peng *et al.* (2017) showed that a hepatitis coronavirus (MHV) infection drives the evolution of a mouse receptor (mCEACAM1a). In this case, this event represented an answer to the intense selective pressure from a lethal infection where MHV uses mCEACAM1a as its host-receptor and changes in critical amino acid residues have led to the emergence of a derived-allele (m*CEACAM1b*) that is much more deficient as MHV receptor (Peng *et al.*, 2017). Consequently, mice that are homozygous for this derived-allele are highly resistant to death from MHV infections (Peng *et al.*, 2017).

The first scientific report on the use of ACE2 as a host-cell entry portal by a coronavirus involved SARS-CoV (Li *et al.*, 2003). If this happened before (some coronavirus use the ACE2 protein as a gateway to the host-cell), either with humans or other species, we currently do not know. Therefore, and considering only what we know about the history of SARS-CoV, it is a short timeframe for us to expect any evolutionary “reaction” from the host with a relatively long life cycle, at least regarding human *ACE2.* In other words, we do not expect to see for the human ACE2 (in relation to both SARS-CoV and SARS-CoV-2) what was reported in the study of Peng *et al.* (2017) for the mouse receptor and MHV. As a consequence of the very recent evolution of SARS-CoV-2 Spike glycoprotein and gain of a cleavage site for furin proteases, in addition to the homogeneity of critical ACE2 binding sites across human populations and other demographic and cultural factors already mentioned above, SARS-CoV-2 obtained a significant edge in the evolutionary arms race, and become a highly infectious and pathogenic coronavirus for humans.

However, it is very well known that subsequent to cell entry, pathogens can be blocked by neutralizing host-antibodies, which appears to be occurring in SARS-CoV-2 infections in humans (Conti *et al.*, 2020). According to the WHO the majority of humans infected by SARS-CoV-2 have mild symptoms or are asymptomatic, despite the fact that they have the same AAs in the ACE2 binding sites as reported here. The mechanisms of how antibodies neutralize a coronavirus infection have already been published (Hofmann *et al.*, 2004; Reguera *et al.*, 2012; Li G *et al.*, 2020), illustrating that an innate agile and efficient host-immune system (like those in young individuals and/or in individuals who do not develop an immunological over-response) is the first weapon to block the infection of opportunistic coronaviruses. Eventual population variability in other genetic systems, which may also be involved in this heterogeneity of COVID-19 symptoms and outcomes, cannot be ruled out. We must also remember that the *ACE2* gene has many polymorphisms outside the regions coding for key binding sites, as mentioned in the last paragraph of the results section. In this way, we cannot rule out that some of these SNPs may also be involved in differential susceptibility for infection, as well as to the differences in the symptoms and outcomes observed across human populations.

On the other hand, the differences in these 30 important ACE2 binding sites of interaction with SARS-CoV-like viruses observed between species and taxonomic groups is striking, probably impacting SARS-Cov-2's ability to bind with ACE2 in some of these species. SARS-CoV-2 capacity to infect other 42 mammalian species was recently predicted based on changes at positions 31, 35, 38, 82, and 353 in the ACE2 orthologues when compared with the human ACE2 (Luan *et al.*, 2020). These authors suggested that 76% of the 42 mammal species investigated, including pets (dog, *Canis lupus familiaris*, and cat, *Felis catus*), horse (*Equus caballus*), cattle (*Bos taurus*), and sheep (*Ovis aries*), may bind to S protein of SARS-CoV-2. The idea of a “generalist’ coronavirus, capable of infecting other species of mammals (pig, ferret, cat, monkey) had also been argued by other authors (Wan *et al.*, 2020), despite of the differences in their correspondent ACE2 orthologues.

Here, we have expanded the comparative analysis to more species and sites of interaction between ACE2 orthologues and SARS-CoV-like viruses. In sum, our results show that ACE2 murine species have only 56% of identity with human ACE2, and have also several critical taxon-specific changes that explain the barrier to infection with SARS-CoV-2. For the other species and taxon investigated here, however, the ability to block (or not) the infection to SARS-CoV-2 via this receptor is not easy to predict. At the moment, preliminary results indicate that a macaque (*Macaca mulatta*) inoculated with SARS-CoV-2 recapitulates moderate respiratory disease observed in the majority of human cases (Munster *et al.*, 2020). Note that *Macaca mulatta*, similar to other species of the family Cercopithecidae, has only one change (R559K) relative to human and other species of the family Hominidae (*Pan troglodytes, Pan paniscus* and *Gorilla gorilla;* Figure 2). We also found a set of AA changes in the ACE2 of NWm species, which can characterize different patterns of binding to SARS-CoV-2, making them potentially less susceptible to the infection than humans and great apes. Earlier, one *in vivo* study (Greenough *et al.*, 2005) demonstrated SARS-CoV infection outcome in *Callithrix sp*., however, the pattern of virus replication and the potential risk for infection in nature for this and other NWm species has never been demonstrated. Therefore, *Callithrix sp.,* although being a good model for MERS-CoV infection (McAuliffe *et al.*, 2004; Van Doremalen and Munster, 2015), it has not been established as an animal model to SARS-CoV infection. Note that MERS-CoV uses another host-cell entry receptor, DPP4, rather than ACE2.

When more *in vitro* and *in vivo* functional studies are performed, we will know what is the minimum AA combination in the ACE2 binding sites and in other critical regions of the receptor that is indispensable for an active connection with SARS-CoV-2 S glycoprotein. Until then, the predictions are speculative, but they can bring subsidies for future studies and strategies to contain the infection and its harmful consequences. Here, we suggest that there are not many ACE2 orthologues with tropism for SARS-CoV-2, precisely because of its ability, shaped by natural selection, to use *Homo sapiens* ACE2 as an efficient and successful gateway to the host-cell. To our knowledge, there is only one preliminary *in vivo* study to test the susceptibility of animals to SARS-CoV-2 (Shi *et al.*, 2020). These authors intra-nasally inoculated ferrets, cats, dogs, pigs, chickens, and ducks with a dosage of SARS-CoV-2, and euthanized them some days post inoculation. They reported that ferrets and cats are highly susceptible to SARS-CoV-2, dogs have low susceptibility, and livestock including pigs, chickens, and ducks are not susceptible to the SARS-CoV-2. On the other hand, there are no scientific record up to date of natural infection by SARS-CoV-2 in animals living in anthropogenic environments (only rare press reports that described that cats, as well as tigers and lions from the Bronx Zoo in New York City were infected), an observation that challenges previous predictions that this coronavirus would infect a wide range of species (Luan *et al.*, 2020; Wan *et al.*, 2020). The absence of a veterinary epidemiological emergency during the current human COVID-19 pandemic, at least considering pets, also challenges preliminary results, which indicated a high susceptibility of cats to SARS-CoV-2 (Shi *et al.*, 2020). If there is such a susceptibility to SARS-CoV-2 infection to these and other animals, it appears that it is much more restricted than that observed for humans.

It is also known that viruses that take advantage of receptors with conserved AA sequence have a broad host range (Woolhouse *et al.*, 2012). It is worth recalling that ACE2 has conserved the AA sequence in crucial binding sites within *Homo sapiens*, but not between placental mammal species investigated here. In other words, the high mutation rate SARS-CoV-2 may provide alternatives to infect one of several species, but when a high tropism with the host cell is found, it can be predicted that the binding region has little (if any) variation within the target species.

Our findings reveal also that an essential part of this taxon-specific ACE2 diversity can even be attributed to the positive natural selection, since it is a membrane protein related to the water intake, blood pressure control, and other cardiovascular conditions. Besides that, a common trade-off of adapting to novel hosts is that the performance on the original host is reduced (Longdon *et al.*, 2014). In other words, SARS-CoV-2 is a coronavirus with high performance to infect *Homo sapiens* individuals, from any population. In contrast, it could not infect, at least with the same efficiency, other species, as well as jump and re-infect the original or intermediate phylogenetically distant hosts (bats and/or pangolins). New mutations and/or recombination can turn SARS-CoV-2 into a derived-coronavirus capable of breaking down species barriers again, feeding the endless cycle of the arms race between host and pathogen.

On the other hand, closely related species may have similar levels of susceptibility, regardless of their distance from the pathogen's natural host (Longdon *et al.*, 2014). Thus, due to the homology that ACE2 orthologues of the great apes have with the human ACE2 regarding the 30 binding sites investigated here, perhaps, only they are naturally and equally susceptible to develop symptoms of COVID-19, a concern that has already been demonstrated by members of the “Great Ape Health Consortium” (Gillespie and Leendertz, 2020).

## Conclusion

Our comparative analysis of the ACE2 coding region from 70 placental mammal species, revealed many variable sites. Of these, we selected 30 sites with records to be relevant for interaction with SARS-CoV-like S glycoproteins. For three of them, there is a significant signal of the action of the Darwinian (positive) selection. We observed extraordinary conservation within *Homo sapiens* and relatively high variation among placental mammal species. Our data indicate that SARS-CoV-2 has similar potential to infect any human population, already corroborated by the pandemic character of COVID-19. We can suggest that any difference in the rate of infection and mortality of this pathogen/disease among humans is related to other factors such as comorbidities of the infected individuals, cultural practices, demographic density, capacity of the country's health systems, suppression or mitigation measures, in addition to differences in the SARS-CoV-2 strains, as well as the effective innate immune response from infected individuals. The involvement of other genetic pathways, in addition to the ACE2 protein and the immune system, cannot be ruled out either, but only the progress of the studies will be able to identify them, as well as their eventual role in the COVID-19 human pandemic.

In conclusion, SARS-CoV-2 is highly specialized for infecting humans of all populations, *i.e.* it is a “human-population generalist coronavirus”, at least considering its gateway to the cell, the ACE2. In contrast, it is not a “generalist” coronavirus with the ability to infect, naturally and easily, a range of other species, including pets.

## Supplementary Material

Table S1 Placental mammal species analyzed.

Table S2 Evolutionary rates estimated considering 70 *ACE2* placental mammal orthologues.

Table S3 Bayesian Empirical Bayes (BEB) analysis indicating sites with high probability (> 95%) to be under positive selection

Table S4 Bayes Empirical Bayes (BEB). General results. Signal peptide (1-17), extracellular domain (residues 18-740), transmembrane domain (741-761), and cytoplasmatic domain (761-805). See ω column.

Figure S1 Highlighted variable sites in placental mammalian ACE2 protein in the interaction with SARS-CoV-2.
