# Supplementary material for: ACE2 diversity in placental mammals reveals the evolutionary strategy of SARS-CoV-2
Source: Genet Mol Biol. 2020 Jun 8;43(2):e20200104. doi: 10.1590/1678-4685-GMB-2020-0104 (PMC7278419; doi:10.1590/1678-4685-GMB-2020-0104)
Supplement: Supplementary file 1 [file 1415-4757-GMB-43-2-e20200104-suppl1.pdf]

Supplementary Material to "ACE2 diversity in placental mammals reveals the evolutionary strategy of SARS-CoV-2"

Table S1 - Placental mammal species analyzed.

ORDER	SPECIES	COMMON NAME	REFERENCE ACE2	LENGTH
Carnivora	*Ailuropoda melanoleuca*	giant panda	XM_019793431.1	805
Carnivora	*Canis lupus familiaris*	dog	NM_001165260.1	804
Carnivora	*Enhydra lutris kenyoni*	sea otter	XM_022518370.1	805
Carnivora	*Eumetopias jubatus*	Steller sea lion	XM_028115021.1	806
Carnivora	*Felis catus*	domestic cat	XM_023248796.1	807
Carnivora	*Lagenorhynchus obliquidens*	Pacific white-sided dolphin	XM_027095797.1	804
Carnivora	*Mustela putorius furo*	domestic ferret	NM_001310190.1	805
Carnivora	*Paguma larvata*	Civet	AY881174.1	805
Carnivora	*Panthera tigris altaica*	Amur tiger	XM_007090080.2	797
Carnivora	*Vulpes vulpes*	red fox	XM_025986727.1	804
Cetartiodactyla	*Bos indicus*	zebu cattle	XM_019956161.1	804
Cetartiodactyla	*Bos mutus*	wild yak	XM_005903111.1	804
Cetartiodactyla	*Bos taurus*	cattle	XM_005228429.4	803
Cetartiodactyla	*Bubalus bubalis*	water buffalo	XM_006041540.2	803
Cetartiodactyla	*Camelus bactrianus*	Bactrian camel	XM_010968001.1	805
Cetartiodactyla	*Camelus dromedarius*	Arabian camel	XM_010993415.2	805
Cetartiodactyla	*Camelus ferus*	Wild Bactrian camel	XM_006194201.1	805
Cetartiodactyla	*Capra hircus*	goat	NM_001290107.1	804
Cetartiodactyla	*Lipotes vexillifer*	Yangtze River dolphin	XM_007466327.1	804
Cetartiodactyla	*Ovis aries*	sheep	XM_012106267.3	804
Cetartiodactyla	*Sus scrofa*	pig	NM_001123070.1	805
Chiroptera	*Desmodus rotundus*	common vampire bat	XM_024569930	804
Chiroptera	*Hipposideros armiger*	great roundleaf bat	XM_019667391.1	806
Chiroptera	*Miniopterus natalensis*	NA	XM_016202967.1	804
Chiroptera	*Myotis brandtii*	Brandts bat	XM_014544296.1	799
Chiroptera	*Myotis davidii*	NA	XM_015571433.1	799
Chiroptera	*Myotis lucifugus*	little brown bat	XM_023753671.1	799
Chiroptera	*Phyllostomus discolor*	pale spear-nosed bat	XM_028522516	804
Chiroptera	*Pteropus alecto*	black flying fox	XM_006911647.1	805
Chiroptera	*Pteropus vampyrus*	large flying fox	XM_011362973.2	804

ORDER	SPECIES	COMMON NAME	REFERENCE ACE2	LENGTH
Chiroptera	*Rousettus aegyptiacus*	Egyptian rousette	XM_016118926.1	805
Eulipotyphla	*Erinaceus europaeus*	western European hedgehog	XM_007538608.2	804
Lagomorpha	*Oryctolagus cuniculus*	rabbit	XM_002719845.3	805
Macroscelidea	*Elephantulus edwardii*	Cape elephant shrew	XM_006892395	798
Perissodactyla	*Equus caballus*	horse	XM_001490191.5	805
Perissodactyla	*Equus przewalskii*	Przewalskis horse	XM_008544773.1	805
Pholidota	*Manis javanica*	Malayan pangolin	XM_017650257.1	805
Primates	*Aotus nancymaae*	Mas night monkey	XM_012434682.2	805
Primates	*Callithrix jacchus*	white-tufted-ear marmoset	XM_008988993	805
Primates	*Carlito syrichta*	Philippine tarsier	XM_008064619	805
Primates	*Cebus capucinus imitator*	NA	XM_017512376	805
Primates	*Cercocebus atys*	sooty mangabey	XM_012035808	805
Primates	*Chlorocebus sabaeus*	green monkey	XM_007991113	805
Primates	*Gorilla gorilla*	western gorilla	XM_019019204.1	805
Primates	*Homo sapiens*	human	NM_001371415.1	805
Primates	*Macaca fascicularis*	crab-eating macaque	XM_011545549	805
Primates	*Macaca mulatta*	Rhesus monkey	NM_001135696	805
Primates	*Macaca nemestrina*	pig-tailed macaque	XM_011735203	805
Primates	*Mandrillus leucophaeus*	drill	XM_011995533	805
Primates	*Microcebus murinus*	gray mouse lemur	XM_020285237.1	851
Primates	*Otolemur garnettii*	small-eared galago	XM_003791864.2	805
Primates	*Pan paniscus*	pygmy chimpanzee	XM_008974180.1	805
Primates	*Pan troglodytes*	chimpanzee	XM_016942979	805
Primates	*Papio anubis*	olive baboon	XM_021933040	805
Primates	*Piliocolobus tephrosceles*	Ugandan red Colobus	XM_023199053	805
Primates	*Propithecus coquereli*	Coquerels sifaka	XM_012638731.1	826
Primates	*Rhinopithecus roxellana*	golden snub-nosed monkey	XM_010366065	805
Primates	*Saimiri boliviensis*	Bolivian squirrel monkey	XM_010336623	805
Primates	*Sapajus apella*	Tufted capuchi	XM_032285963.1	805
Primates	*Theropithecus gelada*	gelada	XM_025372062	805
Rodentia	*Cavia porcellus*	domestic guinea pig	XM_023562040	813
Rodentia	*Cricetulus griseus*	Chinese hamster	XM_003503235	805
Rodentia	*Dipodomys ordii*	Ords kangaroo rat	XM_013032118	805
Rodentia	*Marmota flaviventris*	yellow-bellied marmot	XM_027946507	817

ORDER	SPECIES	COMMON NAME	REFERENCE ACE2	LENGTH
Rodentia	*Marmota marmota marmota*	Alpine marmot	XM_015488054	817
Rodentia	*Mus caroli*	Ryukyu mouse	XM_021153479	805
Rodentia	*Mus musculus*	house mouse	NM_027286	805
Rodentia	*Mus pahari*	shrew mouse	XM_021188276	805
Rodentia	*Rattus norvegicus*	Norway rat	NM_001012006	805
Scandentia	*Tupaia chinensis*	Chinese tree shrew	XM_006164692	805
