# Supplementary material for: ACE2 diversity in placental mammals reveals the evolutionary strategy of SARS-CoV-2
Source: Genet Mol Biol. 2020 Jun 8;43(2):e20200104. doi: 10.1590/1678-4685-GMB-2020-0104 (PMC7278419; doi:10.1590/1678-4685-GMB-2020-0104)
Supplement: Supplementary file 2 [file 1415-4757-GMB-43-2-e20200104-suppl2.pdf]

Supplementary Material to "ACE2 diversity in placental mammals reveals the evolutionary strategy of SARS-CoV-2"

Table S2 - Evolutionary rates estimated considering 70 *ACE2* placental mammal orthologues.

Site Model	dN/dS	Estimated parameters	ℓ	*p*-value
M1: neutral	0,3658	p0= 0.70382, (p1= 0.29618)		
		(ω0= 0.09888), (ω1= 1.00000)	-31476,87406	
M2a: selection	0,451	p0=0.68587, p1= 0.27167, (p2= 0.04246)		
		(ω0= 0.10125), (ω1= 1.00000), ω2=2.58680	-31377,15314	$p<0{,}001$

p0= proportion of sites where ω< 1; p1= proportion of sites where ω = 1 and p2= proportion of sites where ω> 1 (selection models only); ω0< 1 (negative selection), ω1= 1 (neutral selection) and ω2 >1 (positive selection); Likelihood ratio tests were performed between neutral model (M1a), and model that identify positive selection (M2a). In the comparisons, M1a *vs* M2a has 2 degrees of freedom (df=2). Parentheses indicate fixed parameters.
