# Supplementary material for: ACE2 diversity in placental mammals reveals the evolutionary strategy of SARS-CoV-2
Source: Genet Mol Biol. 2020 Jun 8;43(2):e20200104. doi: 10.1590/1678-4685-GMB-2020-0104 (PMC7278419; doi:10.1590/1678-4685-GMB-2020-0104)
Supplement: Supplementary file 3 [file 1415-4757-GMB-43-2-e20200104-suppl3.pdf]

Supplementary Material to “ACE2 diversity in placental mammals reveals the evolutionary strategy of SARS-CoV-2”

Table S3 - Bayesian Empirical Bayes (BEB) analysis indicating sites with high probability (> 95%) to be under positive selection.

Site	Probability of being under positive selection	Post mean +- SE for ω
24	0.985	2.231 +- 0.151
34	0.999	2.249 +- 0.040
91	1.000	2.250 +- 0.003
93	0.954	2.193 +- 0.262
212	1.000	2.249 +- 0.026
228	0.993	2.241 +- 0.105
231	0.994	2.243 +- 0.093
251	0.969	2.211 +- 0.218
255	0.985	2.232 +- 0.151
286	0.951	2.189 +- 0.269
301	0.999	2.248 +- 0.048
387	1.000	2.250 +- 0.011
559	0.990	2.237 +- 0.127
568	0.971	2.214 +- 0.209
607	0.987	2.234 +- 0.142
653	0.994	2.243 +- 0.095
657	0.998	2.248 +- 0.052
658	1.000	2.250 +- 0.005
671	1.000	2.250 +- 0.014
675	0.980	2.225 +- 0.175
689	0.999	2.249 +- 0.039
732	1.000	2.250 +- 0.008
