# Supplementary material for: ACE2 diversity in placental mammals reveals the evolutionary strategy of SARS-CoV-2
Source: Genet Mol Biol. 2020 Jun 8;43(2):e20200104. doi: 10.1590/1678-4685-GMB-2020-0104 (PMC7278419; doi:10.1590/1678-4685-GMB-2020-0104)
Supplement: Supplementary file 4 [file 1415-4757-GMB-43-2-e20200104-suppl4.pdf]

Supplementary Material to "ACE2 diversity in placental mammals reveals the evolutionary strategy of SARS-CoV-2"

Table S4 - Bayes Empirical Bayes (BEB) general results. Signal peptide (1-17), extracellular domain (residues 18-740), transmembrane domain (741 -761), and cytoplasmatic domain (761-805). See ω column.

	Site	W1	W2	W3	W		Site	W1	W2	W3	W
Signal peptide	**1**	1	0.00000	0.00000	0.10215	*Angiotensin-converting enzyme 2 extracellular domain*	**403**	0.99995	0.00005	0.00000	0.10215
	**2**	0.02678	0.96877	0.00446	1		**404**	0.99864	0.00136	0.00000	0.10215
	**3**	0.03837	0.96139	0.00024	1		**405**	0.99988	0.00012	0.00000	0.10215
	**4**	0.99731	0.00269	0.00000	0.10215		**406**	0.99999	0.00001	0.00000	0.10215
	**5**	0.00000	0.78159	0.21841	1		**407**	0.99542	0.00458	0.00000	0.10215
	**6**	0.99489	0.00511	0.00000	0.10215		**408**	0.99999	0.00001	0.00000	0.10215
	**7**	0.99368	0.00632	0.00000	0.10215		**409**	0.99723	0.00277	0.00000	0.10215
	**8**	0.70401	0.29598	0.00001	0.10215		**410**	0.99977	0.00023	0.00000	0.10215
	**9**	0.99362	0.00638	0.00000	0.10215		**411**	0.99968	0.00032	0.00000	0.10215
	**10**	0.99948	0.00052	0.00000	0.10215		**412**	0.95922	0.04078	0.00000	0.10215
	**11**	0.98979	0.01021	0.00000	0.10215		**413**	0.99794	0.00206	0.00000	0.10215
	**12**	0.93414	0.06586	0.00000	0.10215		**414**	0.99986	0.00014	0.00000	0.10215
	**13**	0.98606	0.01394	0.00000	0.10215		**415**	0.99955	0.00045	0.00000	0.10215
	**14**	0.99896	0.00104	0.00000	0.10215		**416**	0.00010	0.99915	0.00075	1
	**15**	0.21743	0.78256	0.00001	1		**417**	0.99985	0.00015	0.00000	0.10215

	**16**	0.99818	0.00182	0.00000	0.10215		**418**	0.99752	0.00248	0.00000	0.10215
	**17**	0.99973	0.00027	0.00000	0.10215		**419**	0.99991	0.00009	0.00000	0.10215
Angiotensin-converting enzyme 2 extracellular domain	**18**	0.99603	0.00397	0.00000	0.10215		**420**	0.00000	0.66347	0.33653	1
	**19**	0.05376	0.94502	0.00122	1		**421**	0.96968	0.03032	0.00000	0.10215
	**20**	0.00008	0.98768	0.01224	1		**422**	0.99996	0.00004	0.00000	0.10215
	**21**	0.00000	0.98744	0.01255	1		**423**	0.99979	0.00021	0.00000	0.10215
	**22**	0.99993	0.00007	0.00000	0.10215		**424**	0.99679	0.00321	0.00000	0.10215
	**23**	0.97862	0.02138	0.00000	0.10215		**425**	0.33487	0.66492	0.00022	1
	**24**	0.00000	0.01801	0.98199	2,56612		**426**	0.00000	0.85601	0.14399	1
	**25**	0.65251	0.34747	0.00002	0.10215		**427**	0.95761	0.04239	0.00000	0.10215
	**26**	0.85409	0.14591	0.00000	0.10215		**428**	0.99995	0.00005	0.00000	0.10215
	**27**	0.00002	0.99209	0.00789	1		**429**	0.00000	0.49034	0.50966	2,56612
	**28**	0.99995	0.00005	0.00000	0.10215		**430**	0.99992	0.00008	0.00000	0.10215
	**29**	0.99796	0.00204	0.00000	0.10215		**431**	0.99999	0.00001	0.00000	0.10215
	**30**	0.00315	0.99680	0.00005	1		**432**	0.00095	0.99897	0.00009	1
	**31**	0.00001	0.99933	0.00066	1		**433**	0.99042	0.00958	0.00000	0.10215
	**32**	0.99995	0.00005	0.00000	0.10215		**434**	0.99986	0.00014	0.00000	0.10215
	**33**	0.99992	0.00008	0.00000	0.10215		**435**	0.05842	0.94156	0.00001	1

	**34**	0.00000	0.00105	0.99895	2,56612		**436**	0.99903	0.00097	0.00000	0.10215
	**35**	0.79593	0.20407	0.00000	0.10215		**437**	0.99999	0.00001	0.00000	0.10215
	**36**	0.99741	0.00259	0.00000	0.10215		**438**	0.99825	0.00175	0.00000	0.10215
	**37**	0.99999	0.00001	0.00000	0.10215		**439**	0.96557	0.03442	0.00001	0.10215
	**38**	0.49442	0.50558	0.00000	1		**440**	0.95944	0.04056	0.00000	0.10215
	**39**	0.83764	0.16227	0.00009	0.10215		**441**	0.99999	0.00001	0.00000	0.10215
	**40**	0.00002	0.98618	0.01380	1		**442**	0.99939	0.00061	0.00000	0.10215
	**41**	0.00081	0.99849	0.00070	1		**443**	0.99986	0.00014	0.00000	0.10215
	**42**	0.48810	0.51184	0.00006	1		**444**	0.99948	0.00052	0.00000	0.10215
	**43**	0.42642	0.57357	0.00000	1		**445**	0.78283	0.21717	0.00000	0.10215
	**44**	0.03240	0.96422	0.00338	1		**446**	0.99668	0.00332	0.00000	0.10215
	**45**	0.99976	0.00024	0.00000	0.10215		**447**	0.99724	0.00276	0.00000	0.10215
	**46**	0.99996	0.00004	0.00000	0.10215		**448**	0.99031	0.00969	0.00000	0.10215
	**47**	0.90859	0.09141	0.00000	0.10215		**449**	0.99965	0.00035	0.00000	0.10215
	**48**	0.99493	0.00507	0.00000	0.10215		**450**	0.96036	0.03963	0.00001	0.10215
	**49**	0.00197	0.99801	0.00002	1		**451**	0.99910	0.00090	0.00000	0.10215
	**50**	0.97093	0.02907	0.00000	0.10215		**452**	0.99993	0.00007	0.00000	0.10215
	**51**	0.99999	0.00001	0.00000	0.10215		**453**	0.99996	0.00004	0.00000	0.10215

	**52**	0.98944	0.01056	0.00000	0.10215		**454**	0.97632	0.02368	0.00000	0.10215
	**53**	1	0.00000	0.00000	0.10215		**455**	1	0.00000	0.00000	0.10215
	**54**	0.99999	0.00001	0.00000	0.10215		**456**	0.99703	0.00297	0.00000	0.10215
	**55**	0.99563	0.00437	0.00000	0.10215		**457**	0.99999	0.00001	0.00000	0.10215
	**56**	0.89717	0.10283	0.00000	0.10215		**458**	0.99999	0.00001	0.00000	0.10215
	**57**	0.99999	0.00001	0.00000	0.10215		**459**	0.96788	0.03211	0.00001	0.10215
	**58**	1	0.00000	0.00000	0.10215		**460**	0.99746	0.00254	0.00000	0.10215
	**59**	0.00000	0.78719	0.21281	1		**461**	0.96788	0.03211	0.00001	0.10215
	**60**	0.99594	0.00406	0.00000	0.10215		**462**	1	0.00000	0.00000	0.10215
	**61**	0.99620	0.00380	0.00000	0.10215		**463**	0.99979	0.00021	0.00000	0.10215
	**62**	1	0.00000	0.00000	0.10215		**464**	0.99994	0.00006	0.00000	0.10215
	**63**	0.99636	0.00364	0.00000	0.10215		**465**	0.00005	0.99926	0.00069	1
	**64**	0.00000	0.98659	0.01341	1		**466**	0.99976	0.00024	0.00000	0.10215
	**65**	0.97868	0.02132	0.00000	0.10215		**467**	0.97354	0.02646	0.00000	0.10215
	**66**	0.00001	0.99429	0.00570	1		**468**	0.99947	0.00053	0.00000	0.10215
	**67**	0.00000	0.94670	0.05330	1		**469**	0.99859	0.00141	0.00000	0.10215
	**68**	0.00405	0.99583	0.00012	1		**470**	0.96739	0.03261	0.00000	0.10215
	**69**	0.39636	0.60257	0.00107	1		**471**	0.51936	0.48064	0.00000	0.10215

**70**	0.98528	0.01472	0.00000	0.10215	**472**	0.48085	0.51908	0.00007	1
**71**	0.01717	0.98223	0.00060	1	**473**	0.99493	0.00507	0.00000	0.10215
**72**	0.99995	0.00005	0.00000	0.10215	**474**	0.99597	0.00403	0.00000	0.10215
**73**	0.99430	0.00570	0.00000	0.10215	**475**	0.00022	0.99932	0.00047	1
**74**	0.99924	0.00076	0.00000	0.10215	**476**	0.99864	0.00136	0.00000	0.10215
**75**	0.97003	0.02997	0.00000	0.10215	**477**	0.96676	0.03323	0.00001	0.10215
**76**	0.46905	0.53088	0.00007	1	**478**	0.99493	0.00507	0.00000	0.10215
**77**	0.69358	0.30634	0.00008	0.10215	**479**	0.99630	0.00370	0.00000	0.10215
**78**	0.01012	0.98979	0.00009	1	**480**	1	0.00000	0.00000	0.10215
**79**	0.00000	0.23458	0.76542	2,56612	**481**	0.99999	0.00001	0.00000	0.10215
**80**	0.98752	0.01248	0.00000	0.10215	**482**	0.96682	0.03317	0.00001	0.10215
**81**	0.21110	0.78888	0.00002	1	**483**	0.86425	0.13575	0.00000	0.10215
**82**	0.00000	0.48804	0.51196	2,56612	**484**	0.99093	0.00907	0.00000	0.10215
**83**	0.95644	0.04356	0.00000	0.10215	**485**	0.99994	0.00006	0.00000	0.10215
**84**	0.05166	0.94705	0.00129	1	**486**	0.99989	0.00011	0.00000	0.10215
**85**	0.40215	0.59682	0.00104	1	**487**	0.99991	0.00009	0.00000	0.10215
**86**	0.00001	0.99120	0.00879	1	**488**	0.97435	0.02565	0.00000	0.10215
**87**	0.00261	0.99732	0.00007	1	**489**	0.99999	0.00001	0.00000	0.10215

	**88**	0.99943	0.00057	0.00000	0.10215		**490**	0.99962	0.00038	0.00000	0.10215
	**89**	0.00269	0.99478	0.00252	1		**491**	0.00000	0.91016	0.08984	1
	**90**	0.08631	0.91368	0.00000	1		**492**	0.94910	0.05090	0.00000	0.10215
	**91**	0.00000	0.00001	0.99999	2,56612		**493**	0.99998	0.00002	0.00000	0.10215
	**92**	0.00000	0.95796	0.04204	1		**494**	0.99993	0.00007	0.00000	0.10215
	**93**	0.00000	0.14520	0.85480	2,56612		**495**	0.99999	0.00001	0.00000	0.10215
	**94**	0.99993	0.00007	0.00000	0.10215		**496**	0.99887	0.00113	0.00000	0.10215
	**95**	0.11417	0.88555	0.00027	1		**497**	0.99948	0.00052	0.00000	0.10215
	**96**	0.99937	0.00063	0.00000	0.10215		**498**	0.99973	0.00027	0.00000	0.10215
	**97**	0.99789	0.00211	0.00000	0.10215		**499**	0.99999	0.00001	0.00000	0.10215
	**98**	0.00002	0.97240	0.02758	1		**500**	0.99948	0.00052	0.00000	0.10215
	**99**	0.00000	0.80755	0.19245	1		**501**	0.99987	0.00013	0.00000	0.10215
	**100**	0.99958	0.00042	0.00000	0.10215		**502**	0.00002	0.98413	0.01586	1
	**101**	0.99632	0.00368	0.00000	0.10215		**503**	0.99675	0.00325	0.00000	0.10215
	**102**	0.00307	0.99494	0.00199	1		**504**	0.99825	0.00175	0.00000	0.10215
	**103**	0.80630	0.19370	0.00000	0.10215		**505**	0.99998	0.00002	0.00000	0.10215
	**104**	0.99910	0.00090	0.00000	0.10215		**506**	0.99997	0.00003	0.00000	0.10215
	**105**	0.75319	0.24677	0.00004	0.10215		**507**	0.98953	0.01047	0.00000	0.10215

	**106**	0.86360	0.13634	0.00006	0.10215		**508**	0.99992	0.00008	0.00000	0.10215
	**107**	0.00000	0.96295	0.03705	1		**509**	1	0.00000	0.00000	0.10215
	**108**	0.98734	0.01266	0.00000	0.10215		**510**	0.97830	0.02170	0.00000	0.10215
	**109**	0.55182	0.44786	0.00033	0.10215		**511**	0.99722	0.00278	0.00000	0.10215
	**110**	0.00003	0.99380	0.00617	1		**512**	0.99975	0.00025	0.00000	0.10215
	**111**	0.99683	0.00317	0.00000	0.10215		**513**	0.99994	0.00006	0.00000	0.10215
	**112**	0.99999	0.00001	0.00000	0.10215		**514**	0.99769	0.00231	0.00000	0.10215
	**113**	0.00000	0.94897	0.05103	1		**515**	0.99990	0.00010	0.00000	0.10215
	**114**	0.75270	0.24730	0.00000	0.10215		**516**	0.99683	0.00317	0.00000	0.10215
	**115**	0.00008	0.91865	0.08126	1		**517**	0.99986	0.00014	0.00000	0.10215
	**116**	0.99787	0.00213	0.00000	0.10215		**518**	0.99995	0.00005	0.00000	0.10215
	**117**	0.97646	0.02354	0.00000	0.10215		**519**	0.99974	0.00026	0.00000	0.10215
	**118**	0.00001	0.98492	0.01508	1		**520**	0.99991	0.00009	0.00000	0.10215
	**119**	0.99991	0.00009	0.00000	0.10215		**521**	0.99929	0.00071	0.00000	0.10215
	**120**	0.97939	0.02061	0.00000	0.10215		**522**	0.98065	0.01935	0.00000	0.10215
	**121**	0.22556	0.77444	0.00000	1		**523**	0.99976	0.00024	0.00000	0.10215
	**122**	0.00894	0.99069	0.00038	1		**524**	0.99944	0.00056	0.00000	0.10215
	**123**	1	0.00000	0.00000	0.10215		**525**	0.99995	0.00005	0.00000	0.10215

	**124**	0.99992	0.00008	0.00000	0.10215		**526**	0.88267	0.11732	0.00000	0.10215
	**125**	0.98902	0.01098	0.00000	0.10215		**527**	0.99999	0.00001	0.00000	0.10215
	**126**	0.97929	0.02071	0.00000	0.10215		**528**	0.99978	0.00022	0.00000	0.10215
	**127**	0.99950	0.00050	0.00000	0.10215		**529**	0.99978	0.00022	0.00000	0.10215
	**128**	0.99999	0.00001	0.00000	0.10215		**530**	0.99972	0.00028	0.00000	0.10215
	**129**	0.98847	0.01153	0.00000	0.10215		**531**	0.00000	0.73466	0.26534	1
	**130**	0.99982	0.00018	0.00000	0.10215		**532**	0.00031	0.99805	0.00164	1
	**131**	0.65821	0.34178	0.00000	0.10215		**533**	0.99996	0.00004	0.00000	0.10215
	**132**	0.99843	0.00157	0.00000	0.10215		**534**	0.29974	0.70025	0.00001	1
	**133**	0.98125	0.01875	0.00000	0.10215		**535**	0.99279	0.00721	0.00000	0.10215
	**134**	0.09024	0.90975	0.00001	1		**536**	0.82570	0.17430	0.00000	0.10215
	**135**	0.77391	0.22606	0.00003	0.10215		**537**	0.99992	0.00008	0.00000	0.10215
	**136**	0.00000	0.99839	0.00161	1		**538**	0.94632	0.05367	0.00000	0.10215
	**137**	0.91881	0.08119	0.00000	0.10215		**539**	0.99682	0.00318	0.00000	0.10215
	**138**	0.94983	0.05017	0.00000	0.10215		**540**	0.07295	0.92697	0.00008	1
	**139**	0.97344	0.02656	0.00000	0.10215		**541**	0.99935	0.00065	0.00000	0.10215
	**140**	0.99691	0.00309	0.00000	0.10215		**542**	0.99973	0.00027	0.00000	0.10215
	**141**	0.99893	0.00107	0.00000	0.10215		**543**	0.99999	0.00001	0.00000	0.10215

	**142**	0.00410	0.98413	0.01177	1		**544**	0.99992	0.00008	0.00000	0.10215
	**143**	0.00072	0.97069	0.02859	1		**545**	0.98225	0.01775	0.00000	0.10215
	**144**	0.99979	0.00021	0.00000	0.10215		**546**	0.99937	0.00063	0.00000	0.10215
	**145**	0.81519	0.18481	0.00000	0.10215		**547**	0.99885	0.00115	0.00000	0.10215
	**146**	0.99328	0.00672	0.00000	0.10215		**548**	0.00002	0.99535	0.00462	1
	**147**	0.99967	0.00033	0.00000	0.10215		**549**	0.99532	0.00468	0.00000	0.10215
	**148**	0.99801	0.00199	0.00000	0.10215		**550**	0.99996	0.00004	0.00000	0.10215
	**149**	0.96320	0.03680	0.00000	0.10215		**551**	0.99988	0.00012	0.00000	0.10215
	**150**	0.00000	0.77262	0.22738	1		**552**	0.00002	0.98876	0.01122	1
	**151**	0.99999	0.00001	0.00000	0.10215		**553**	0.99572	0.00428	0.00000	0.10215
	**152**	1	0.00000	0.00000	0.10215		**554**	0.99702	0.00298	0.00000	0.10215
	**153**	0.00055	0.99868	0.00078	1		**555**	0.39019	0.60966	0.00014	1
	**154**	0.00019	0.99919	0.00063	1		**556**	0.00000	0.96632	0.03368	1
	**155**	0.99994	0.00006	0.00000	0.10215		**557**	1	0.00000	0.00000	0.10215
	**156**	0.00051	0.99860	0.00090	1		**558**	0.97971	0.02028	0.00000	0.10215
	**157**	0.99999	0.00001	0.00000	0.10215		**559**	0.00000	0.01323	0.98677	2,56612
	**158**	0.99950	0.00050	0.00000	0.10215		**560**	0.99846	0.00154	0.00000	0.10215
	**159**	0.01040	0.98959	0.00001	1		**561**	0.99981	0.00019	0.00000	0.10215

	**160**	0.00000	0.44403	0.55597	2,56612		**562**	0.97028	0.02972	0.00000	0.10215
	**161**	0.99994	0.00006	0.00000	0.10215		**563**	0.99734	0.00266	0.00000	0.10215
	**162**	0.99904	0.00096	0.00000	0.10215		**564**	0.01951	0.98046	0.00003	1
	**163**	0.99493	0.00507	0.00000	0.10215		**565**	0.99856	0.00144	0.00000	0.10215
	**164**	0.91697	0.08303	0.00000	0.10215		**566**	0.99493	0.00507	0.00000	0.10215
	**165**	0.99493	0.00507	0.00000	0.10215		**567**	0.99976	0.00024	0.00000	0.10215
	**166**	0.99999	0.00001	0.00000	0.10215		**568**	0.00000	0.03607	0.96393	2,56612
	**167**	0.68776	0.31223	0.00001	0.10215		**569**	0.99987	0.00013	0.00000	0.10215
	**168**	0.99493	0.00507	0.00000	0.10215		**570**	0.99772	0.00228	0.00000	0.10215
	**169**	0.99993	0.00007	0.00000	0.10215		**571**	0.97598	0.02402	0.00000	0.10215
	**170**	0.00547	0.99384	0.00069	1		**572**	0.00000	0.96747	0.03253	1
	**171**	0.09281	0.90718	0.00001	1		**573**	0.00333	0.99632	0.00034	1
	**172**	0.99452	0.00548	0.00000	0.10215		**574**	0.99929	0.00071	0.00000	0.10215
	**173**	0.99975	0.00025	0.00000	0.10215		**575**	0.99110	0.00890	0.00000	0.10215
	**174**	0.99952	0.00048	0.00000	0.10215		**576**	0.00000	0.90879	0.09121	1
	**175**	0.99614	0.00386	0.00000	0.10215		**577**	0.76767	0.23233	0.00000	0.10215
	**176**	0.99667	0.00333	0.00000	0.10215		**578**	0.23779	0.76221	0.00000	1
	**177**	0.99995	0.00005	0.00000	0.10215		**579**	1	0.00000	0.00000	0.10215

	**178**	0.99911	0.00089	0.00000	0.10215		**580**	0.87200	0.12800	0.00000	0.10215
	**179**	0.39392	0.60514	0.00094	1		**581**	0.99449	0.00551	0.00000	0.10215
	**180**	0.99991	0.00009	0.00000	0.10215		**582**	0.00000	0.97716	0.02284	1
	**181**	0.99999	0.00001	0.00000	0.10215		**583**	0.99910	0.00090	0.00000	0.10215
	**182**	0.99999	0.00001	0.00000	0.10215		**584**	0.99692	0.00308	0.00000	0.10215
	**183**	0.99989	0.00011	0.00000	0.10215		**585**	0.99909	0.00091	0.00000	0.10215
	**184**	0.99991	0.00009	0.00000	0.10215		**586**	0.00960	0.99036	0.00004	1
	**185**	0.00009	0.99292	0.00699	1		**587**	0.99946	0.00054	0.00000	0.10215
	**186**	0.99699	0.00301	0.00000	0.10215		**588**	0.99994	0.00006	0.00000	0.10215
	**187**	0.99989	0.00011	0.00000	0.10215		**589**	0.97007	0.02993	0.00000	0.10215
	**188**	1	0.00000	0.00000	0.10215		**590**	0.99878	0.00122	0.00000	0.10215
	**189**	0.99999	0.00001	0.00000	0.10215		**591**	0.99806	0.00194	0.00000	0.10215
	**190**	1	0.00000	0.00000	0.10215		**592**	0.01817	0.98172	0.00011	1
	**191**	0.99986	0.00014	0.00000	0.10215		**593**	0.17323	0.82663	0.00015	1
	**192**	0.99635	0.00365	0.00000	0.10215		**594**	0.99493	0.00507	0.00000	0.10215
	**193**	0.94756	0.05244	0.00000	0.10215		**595**	0.99684	0.00316	0.00000	0.10215
	**194**	0.97887	0.02113	0.00000	0.10215		**596**	0.96260	0.03740	0.00000	0.10215
	**195**	0.01284	0.98715	0.00001	1		**597**	0.00000	0.97341	0.02659	1

	**196**	0.99989	0.00011	0.00000	0.10215		**598**	0.99622	0.00378	0.00000	0.10215
	**197**	0.97651	0.02349	0.00000	0.10215		**599**	0.99999	0.00001	0.00000	0.10215
	**198**	0.99999	0.00001	0.00000	0.10215		**600**	0.00001	0.99466	0.00534	1
	**199**	0.99988	0.00012	0.00000	0.10215		**601**	0.99959	0.00041	0.00000	0.10215
	**200**	0.99988	0.00012	0.00000	0.10215		**602**	0.99960	0.00040	0.00000	0.10215
	**201**	1	0.00000	0.00000	0.10215		**603**	0.01296	0.98685	0.00020	1
	**202**	0.99990	0.00010	0.00000	0.10215		**604**	0.99924	0.00076	0.00000	0.10215
	**203**	0.99493	0.00507	0.00000	0.10215		**605**	0.99984	0.00016	0.00000	0.10215
	**204**	0.99990	0.00010	0.00000	0.10215		**606**	0.99493	0.00507	0.00000	0.10215
	**205**	0.18546	0.81446	0.00009	1		**607**	0.00000	0.03786	0.96214	2,56612
	**206**	0.99991	0.00009	0.00000	0.10215		**608**	0.91508	0.08492	0.00000	0.10215
	**207**	0.99991	0.00009	0.00000	0.10215		**609**	0.00000	0.89093	0.10907	1
	**208**	0.99941	0.00059	0.00000	0.10215		**610**	0.96502	0.03497	0.00001	0.10215
	**209**	0.00227	0.99728	0.00045	1		**611**	0.24780	0.75219	0.00001	1
	**210**	0.44445	0.55555	0.00000	1		**612**	0.99313	0.00687	0.00000	0.10215
	**211**	0.84070	0.15930	0.00000	0.10215		**613**	0.35665	0.64333	0.00002	1
	**212**	0.00000	0.00037	0.99963	2,56612		**614**	0.00001	0.99573	0.00426	1
	**213**	0.00000	0.97287	0.02713	1		**615**	0.08606	0.91393	0.00001	1

	**214**	0.64828	0.35170	0.00001	0.10215		**616**	0.11184	0.88783	0.00033	1
	**215**	0.99031	0.00969	0.00000	0.10215		**617**	0.32536	0.67462	0.00002	1
	**216**	0.00000	0.81285	0.18715	1		**618**	0.99995	0.00005	0.00000	0.10215
	**217**	0.99909	0.00091	0.00000	0.10215		**619**	0.99999	0.00001	0.00000	0.10215
	**218**	0.00083	0.99879	0.00038	1		**620**	0.99991	0.00009	0.00000	0.10215
	**219**	0.26603	0.73359	0.00038	1		**621**	0.99994	0.00006	0.00000	0.10215
	**220**	0.00000	0.42366	0.57634	2,56612		**622**	0.99999	0.00001	0.00000	0.10215
	**221**	0.99637	0.00363	0.00000	0.10215		**623**	0.99992	0.00008	0.00000	0.10215
	**222**	0.99785	0.00215	0.00000	0.10215		**624**	0.99644	0.00356	0.00000	0.10215
	**223**	0.15425	0.84575	0.00000	1		**625**	0.99942	0.00058	0.00000	0.10215
	**224**	0.00000	0.99538	0.00462	1		**626**	0.15588	0.84245	0.00167	1
	**225**	0.99999	0.00001	0.00000	0.10215		**627**	0.99974	0.00026	0.00000	0.10215
	**226**	0.99910	0.00090	0.00000	0.10215		**628**	0.99981	0.00019	0.00000	0.10215
	**227**	0.99942	0.00058	0.00000	0.10215		**629**	0.99981	0.00019	0.00000	0.10215
	**228**	0.00000	0.02022	0.97978	2,56612		**630**	0.00000	0.29321	0.70679	2,56612
	**229**	0.04762	0.95216	0.00022	1		**631**	0.00000	0.97292	0.02708	1
	**230**	0.99993	0.00007	0.00000	0.10215		**632**	0.99379	0.00621	0.00000	0.10215
	**231**	0.00000	0.03886	0.96114	2,56612		**633**	0.99893	0.00107	0.00000	0.10215

	**232**	0.93838	0.06162	0.00000	0.10215		**634**	0.00005	0.99941	0.00053	1
	**233**	0.99938	0.00062	0.00000	0.10215		**635**	0.99493	0.00507	0.00000	0.10215
	**234**	0.99920	0.00080	0.00000	0.10215		**636**	0.99988	0.00012	0.00000	0.10215
	**235**	0.99906	0.00094	0.00000	0.10215		**637**	0.87508	0.12492	0.00000	0.10215
	**236**	0.99721	0.00279	0.00000	0.10215		**638**	0.99590	0.00410	0.00000	0.10215
	**237**	0.99990	0.00010	0.00000	0.10215		**639**	0.99999	0.00001	0.00000	0.10215
	**238**	0.97018	0.02982	0.00000	0.10215		**640**	0.99769	0.00231	0.00000	0.10215
	**239**	0.00007	0.99868	0.00125	1		**641**	0.97837	0.02163	0.00000	0.10215
	**240**	0.99977	0.00023	0.00000	0.10215		**642**	0.22111	0.77807	0.00083	1
	**241**	0.99998	0.00002	0.00000	0.10215		**643**	0.99974	0.00026	0.00000	0.10215
	**242**	0.99990	0.00010	0.00000	0.10215		**644**	0.00000	0.14533	0.85467	2,56612
	**243**	0.99934	0.00066	0.00000	0.10215		**645**	0.88881	0.11116	0.00003	0.10215
	**244**	0.99991	0.00009	0.00000	0.10215		**646**	0.88418	0.11582	0.00000	0.10215
	**245**	0.99994	0.00006	0.00000	0.10215		**647**	0.00103	0.99871	0.00026	1
	**246**	0.00000	0.72863	0.27137	1		**648**	0.99986	0.00014	0.00000	0.10215
	**247**	0.99999	0.00001	0.00000	0.10215		**649**	0.99929	0.00071	0.00000	0.10215
	**248**	0.99799	0.00201	0.00000	0.10215		**650**	0.98847	0.01153	0.00000	0.10215
	**249**	0.99999	0.00001	0.00000	0.10215		**651**	0.99996	0.00004	0.00000	0.10215

	**250**	0.00894	0.99104	0.00002	1		**652**	0.99991	0.00009	0.00000	0.10215
	**251**	0.00000	0.04017	0.95983	2,56612		**653**	0.00000	0.00671	0.99329	2,56612
	**252**	0.99953	0.00047	0.00000	0.10215		**654**	0.94393	0.05607	0.00000	0.10215
	**253**	0.99965	0.00035	0.00000	0.10215		**655**	0.99995	0.00005	0.00000	0.10215
	**254**	0.93674	0.06326	0.00001	0.10215		**656**	0.00000	0.31527	0.68473	2,56612
	**255**	0.00000	0.02186	0.97814	2,56612		**657**	0.00000	0.00190	0.99810	2,56612
	**256**	0.97447	0.02553	0.00000	0.10215		**658**	0.00000	0.00001	0.99999	2,56612
	**257**	0.97129	0.02871	0.00000	0.10215		**659**	0.99465	0.00535	0.00000	0.10215
	**258**	0.99915	0.00085	0.00000	0.10215		**660**	0.00000	0.99720	0.00279	1
	**259**	0.66603	0.33397	0.00000	0.10215		**661**	0.86992	0.13008	0.00001	0.10215
	**260**	0.99981	0.00019	0.00000	0.10215		**662**	0.00027	0.99806	0.00168	1
	**261**	0.73199	0.26797	0.00004	0.10215		**663**	0.00214	0.99778	0.00008	1
	**262**	0.99917	0.00083	0.00000	0.10215		**664**	0.00000	0.63367	0.36633	1
	**263**	0.99965	0.00035	0.00000	0.10215		**665**	0.99994	0.00006	0.00000	0.10215
	**264**	0.99991	0.00009	0.00000	0.10215		**666**	0.00014	0.99698	0.00288	1
	**265**	0.99998	0.00002	0.00000	0.10215		**667**	0.00000	0.92432	0.07568	1
	**266**	0.99803	0.00197	0.00000	0.10215		**668**	0.00005	0.99951	0.00044	1
	**267**	0.99977	0.00023	0.00000	0.10215		**669**	0.00117	0.99879	0.00004	1

	**268**	0.99995	0.00005	0.00000	0.10215		**670**	0.99934	0.00066	0.00000	0.10215
	**269**	1	0.00000	0.00000	0.10215		**671**	0.00000	0.00007	0.99993	2,56612
	**270**	1	0.00000	0.00000	0.10215		**672**	0.98943	0.01057	0.00000	0.10215
	**271**	0.99493	0.00507	0.00000	0.10215		**673**	0.00000	0.99226	0.00774	1
	**272**	0.99996	0.00004	0.00000	0.10215		**674**	0.84342	0.15658	0.00000	0.10215
	**273**	0.99990	0.00010	0.00000	0.10215		**675**	0.00000	0.02339	0.97661	2,56612
	**274**	0.99995	0.00005	0.00000	0.10215		**676**	0.77035	0.22965	0.00000	0.10215
	**275**	0.99493	0.00507	0.00000	0.10215		**677**	0.16823	0.83116	0.00061	1
	**276**	0.99914	0.00086	0.00000	0.10215		**678**	0.99989	0.00011	0.00000	0.10215
	**277**	0.99993	0.00007	0.00000	0.10215		**679**	0.00005	0.99695	0.00300	1
	**278**	0.99705	0.00295	0.00000	0.10215		**680**	0.99865	0.00135	0.00000	0.10215
	**279**	0.99949	0.00051	0.00000	0.10215		**681**	0.99977	0.00023	0.00000	0.10215
	**280**	0.00000	0.69098	0.30902	1		**682**	0.00002	0.99852	0.00146	1
	**281**	0.98543	0.01457	0.00000	0.10215		**683**	0.99974	0.00026	0.00000	0.10215
	**282**	0.67652	0.32347	0.00001	0.10215		**684**	0.00007	0.99846	0.00148	1
	**283**	0.47440	0.52559	0.00001	1		**685**	0.99980	0.00020	0.00000	0.10215
	**284**	0.99868	0.00132	0.00000	0.10215		**686**	0.99996	0.00004	0.00000	0.10215
	**285**	0.98226	0.01774	0.00000	0.10215		**687**	0.00000	0.39883	0.60117	2,56612

	**286**	0.00000	0.07530	0.92470	2,56612		**688**	0.99738	0.00262	0.00000	0.10215
	**287**	0.00058	0.99586	0.00356	1		**689**	0.00000	0.00203	0.99797	2,56612
	**288**	0.99991	0.00009	0.00000	0.10215		**690**	0.98714	0.01286	0.00000	0.10215
	**289**	0.99909	0.00091	0.00000	0.10215		**691**	0.00000	0.64629	0.35371	1
	**290**	0.99910	0.00090	0.00000	0.10215		**692**	0.17166	0.82822	0.00013	1
	**291**	0.99999	0.00001	0.00000	0.10215		**693**	0.00200	0.99791	0.00009	1
	**292**	1	0.00000	0.00000	0.10215		**694**	0.00005	0.99658	0.00338	1
	**293**	0.99980	0.00020	0.00000	0.10215		**695**	0.99613	0.00387	0.00000	0.10215
	**294**	0.99996	0.00004	0.00000	0.10215		**696**	0.99962	0.00038	0.00000	0.10215
	**295**	0.00022	0.99971	0.00007	1		**697**	0.99603	0.00397	0.00000	0.10215
	**296**	0.00029	0.99816	0.00155	1		**698**	0.00000	0.97188	0.02812	1
	**297**	1	0.00000	0.00000	0.10215		**699**	0.86554	0.13445	0.00000	0.10215
	**298**	0.00000	0.97958	0.02042	1		**700**	0.99997	0.00003	0.00000	0.10215
	**299**	0.00000	0.99742	0.00258	1		**701**	0.99999	0.00001	0.00000	0.10215
	**300**	0.99945	0.00055	0.00000	0.10215		**702**	0.00000	0.91995	0.08005	1
	**301**	0.00000	0.00313	0.99687	2,56612		**703**	0.99889	0.00111	0.00000	0.10215
	**302**	0.99493	0.00507	0.00000	0.10215		**704**	0.99622	0.00378	0.00000	0.10215
	**303**	0.52612	0.47388	0.00000	0.10215		**705**	0.60448	0.39552	0.00000	0.10215

	**304**	0.99907	0.00093	0.00000	0.10215		**706**	0.00000	0.99972	0.00028	1
	**305**	0.00000	0.36274	0.63726	2,56612		**707**	0.72928	0.27067	0.00005	0.10215
	**306**	0.58170	0.41830	0.00000	0.10215		**708**	0.99817	0.00183	0.00000	0.10215
	**307**	0.99999	0.00001	0.00000	0.10215		**709**	0.00000	0.92595	0.07405	1
	**308**	0.99848	0.00152	0.00000	0.10215		**710**	0.99949	0.00051	0.00000	0.10215
	**309**	0.00614	0.99377	0.00009	1		**711**	0.99992	0.00008	0.00000	0.10215
	**310**	0.99999	0.00001	0.00000	0.10215		**712**	1	0.00000	0.00000	0.10215
	**311**	0.99995	0.00005	0.00000	0.10215		**713**	0.99880	0.00120	0.00000	0.10215
	**312**	0.99999	0.00001	0.00000	0.10215		**714**	0.00001	0.99569	0.00430	1
	**313**	0.99705	0.00295	0.00000	0.10215		**715**	0.99982	0.00018	0.00000	0.10215
	**314**	0.99975	0.00025	0.00000	0.10215		**716**	0.00000	0.84575	0.15425	1
	**315**	0.99963	0.00037	0.00000	0.10215		**717**	0.99668	0.00332	0.00000	0.10215
	**316**	0.06299	0.93686	0.00015	1		**718**	0.99992	0.00008	0.00000	0.10215
	**317**	0.99786	0.00214	0.00000	0.10215		**719**	0.99999	0.00001	0.00000	0.10215
	**318**	0.93997	0.06003	0.00000	0.10215		**720**	0.99699	0.00301	0.00000	0.10215
	**319**	0.99166	0.00834	0.00000	0.10215		**721**	0.00140	0.99790	0.00070	1
	**320**	0.99979	0.00021	0.00000	0.10215		**722**	0.99672	0.00328	0.00000	0.10215
	**321**	0.57589	0.42408	0.00003	0.10215		**723**	0.99999	0.00001	0.00000	0.10215

	**322**	0.00000	0.99871	0.00129	1		**724**	0.99995	0.00005	0.00000	0.10215
	**323**	1	0.00000	0.00000	0.10215		**725**	0.14109	0.85708	0.00182	1
	**324**	0.99996	0.00004	0.00000	0.10215		**726**	0.99992	0.00008	0.00000	0.10215
	**325**	0.00048	0.99482	0.00469	1		**727**	0.99992	0.00008	0.00000	0.10215
	**326**	0.00020	0.99771	0.00208	1		**728**	0.00000	0.92280	0.07720	1
	**327**	0.99974	0.00026	0.00000	0.10215		**729**	0.80969	0.19029	0.00002	0.10215
	**328**	0.99493	0.00507	0.00000	0.10215		**730**	0.99987	0.00013	0.00000	0.10215
	**329**	0.00000	0.79026	0.20974	1		**731**	0.99347	0.00653	0.00000	0.10215
	**330**	0.99722	0.00278	0.00000	0.10215		**732**	0.00000	0.00002	0.99998	2,56612
	**331**	0.99873	0.00127	0.00000	0.10215		**733**	0.80017	0.19981	0.00002	0.10215
	**332**	0.99996	0.00004	0.00000	0.10215		**734**	0.99768	0.00232	0.00000	0.10215
	**333**	0.99649	0.00351	0.00000	0.10215		**735**	0.49928	0.50066	0.00006	1
	**334**	0.99809	0.00191	0.00000	0.10215		**736**	0.00068	0.99645	0.00287	1
	**335**	0.97910	0.02090	0.00000	0.10215		**737**	0.34226	0.65754	0.00020	1
	**336**	0.99908	0.00092	0.00000	0.10215		**738**	0.98428	0.01572	0.00000	0.10215
	**337**	0.00000	0.97049	0.02950	1		**739**	0.31005	0.68990	0.00005	1
	**338**	0.99455	0.00545	0.00000	0.10215		**740**	0.03702	0.96268	0.00030	1
	**339**	0.00067	0.99792	0.00141	1	***memb rane***	**741**	0.42008	0.57992	0.00000	1

	**340**	0.04261	0.95509	0.00231	1		**742**	0.99493	0.00507	0.00000	0.10215
	**341**	0.99941	0.00059	0.00000	0.10215		**743**	0.99666	0.00334	0.00000	0.10215
	**342**	0.99942	0.00058	0.00000	0.10215		**744**	0.99993	0.00007	0.00000	0.10215
	**343**	0.66924	0.33074	0.00002	0.10215		**745**	0.00600	0.99385	0.00015	1
	**344**	0.99893	0.00107	0.00000	0.10215		**746**	0.99995	0.00005	0.00000	0.10215
	**345**	0.99990	0.00010	0.00000	0.10215		**747**	0.99988	0.00012	0.00000	0.10215
	**346**	0.99871	0.00129	0.00000	0.10215		**748**	0.97979	0.02021	0.00000	0.10215
	**347**	0.99986	0.00014	0.00000	0.10215		**749**	0.99991	0.00009	0.00000	0.10215
	**348**	0.99995	0.00005	0.00000	0.10215		**750**	0.99999	0.00001	0.00000	0.10215
	**349**	0.99493	0.00507	0.00000	0.10215		**751**	0.70157	0.29842	0.00001	0.10215
	**350**	0.99999	0.00001	0.00000	0.10215		**752**	0.00000	0.30667	0.69333	2,56612
	**351**	0.98076	0.01924	0.00000	0.10215		**753**	0.83656	0.16344	0.00000	0.10215
	**352**	0.99467	0.00533	0.00000	0.10215		**754**	0.99572	0.00428	0.00000	0.10215
	**353**	0.84511	0.15489	0.00000	0.10215		**755**	0.00730	0.99261	0.00010	1
	**354**	0.00000	0.67987	0.32013	1		**756**	0.99388	0.00612	0.00000	0.10215
	**355**	0.99999	0.00001	0.00000	0.10215		**757**	0.99440	0.00560	0.00000	0.10215
	**356**	0.99974	0.00026	0.00000	0.10215		**758**	0.00002	0.99016	0.00982	1
	**357**	0.99994	0.00006	0.00000	0.10215		**759**	0.00001	0.96428	0.03572	1

	**358**	0.99992	0.00008	0.00000	0.10215		**760**	0.99820	0.00180	0.00000	0.10215
	**359**	0.14690	0.85308	0.00002	1		**761**	0.97386	0.02614	0.00000	0.10215
	**360**	1	0.00000	0.00000	0.10215	***Citoplasmatic***	**762**	0.00001	0.97530	0.02469	1
	**361**	0.99893	0.00107	0.00000	0.10215		**763**	0.01392	0.98590	0.00018	1
	**362**	0.99986	0.00014	0.00000	0.10215		**764**	0.99988	0.00012	0.00000	0.10215
	**363**	0.99999	0.00001	0.00000	0.10215		**765**	0.99992	0.00008	0.00000	0.10215
	**364**	0.99542	0.00458	0.00000	0.10215		**766**	0.97316	0.02684	0.00000	0.10215
	**365**	0.99913	0.00087	0.00000	0.10215		**767**	0.00000	0.99921	0.00079	1
	**366**	1	0.00000	0.00000	0.10215		**768**	0.96403	0.03596	0.00001	0.10215
	**367**	0.99808	0.00192	0.00000	0.10215		**769**	0.18348	0.81650	0.00001	1
	**368**	0.96687	0.03313	0.00000	0.10215		**770**	0.99214	0.00786	0.00000	0.10215
	**369**	0.99974	0.00026	0.00000	0.10215		**771**	0.42629	0.57371	0.00000	1
	**370**	0.99683	0.00317	0.00000	0.10215		**772**	0.00000	0.99150	0.00850	1
	**371**	0.99987	0.00013	0.00000	0.10215		**773**	0.02291	0.97637	0.00072	1
	**372**	0.99981	0.00019	0.00000	0.10215		**774**	0.00002	0.98365	0.01633	1
	**373**	0.99998	0.00002	0.00000	0.10215		**775**	0.00001	0.99189	0.00810	1
	**374**	0.99996	0.00004	0.00000	0.10215		**776**	0.00011	0.99922	0.00068	1
	**375**	0.99999	0.00001	0.00000	0.10215		**777**	0.99934	0.00066	0.00000	0.10215

	**376**	1	0.00000	0.00000	0.10215		**778**	0.99942	0.00058	0.00000	0.10215
	**377**	0.99982	0.00018	0.00000	0.10215		**779**	1	0.00000	0.00000	0.10215
	**378**	0.99990	0.00010	0.00000	0.10215		**780**	0.99966	0.00034	0.00000	0.10215
	**379**	0.99993	0.00007	0.00000	0.10215		**781**	0.99989	0.00011	0.00000	0.10215
	**380**	0.99945	0.00055	0.00000	0.10215		**782**	0.04027	0.95947	0.00027	1
	**381**	0.99991	0.00009	0.00000	0.10215		**783**	0.10549	0.89407	0.00043	1
	**382**	0.99964	0.00036	0.00000	0.10215		**784**	0.01323	0.98658	0.00019	1
	**383**	1	0.00000	0.00000	0.10215		**785**	0.98593	0.01407	0.00000	0.10215
	**384**	0.99987	0.00013	0.00000	0.10215		**786**	0.00011	0.99259	0.00730	1
	**385**	0.99991	0.00009	0.00000	0.10215		**787**	0.00019	0.99957	0.00023	1
	**386**	0.99860	0.00140	0.00000	0.10215		**788**	0.97281	0.02719	0.00000	0.10215
	**387**	0.00000	0.00006	0.99994	2,56612		**789**	0.99087	0.00913	0.00000	0.10215
	**388**	0.99937	0.00063	0.00000	0.10215		**790**	0.99992	0.00008	0.00000	0.10215
	**389**	0.99471	0.00529	0.00000	0.10215		**791**	0.06876	0.93124	0.00000	1
	**390**	0.17172	0.82814	0.00014	1		**792**	0.99999	0.00001	0.00000	0.10215
	**391**	0.99664	0.00336	0.00000	0.10215		**793**	0.00023	0.98351	0.01627	1
	**392**	0.86944	0.13051	0.00005	0.10215		**794**	0.90080	0.09920	0.00000	0.10215
	**393**	0.99923	0.00077	0.00000	0.10215		**795**	0.98817	0.01183	0.00000	0.10215

	**394**	0.45296	0.54704	0.00000	1		**796**	0.97189	0.02811	0.00000	0.10215
	**395**	0.99981	0.00019	0.00000	0.10215		**797**	0.20042	0.79957	0.00000	1
	**396**	0.99995	0.00005	0.00000	0.10215		**798**	0.00000	0.14867	0.85133	2,56612
	**397**	1	0.00000	0.00000	0.10215		**799**	0.99646	0.00354	0.00000	0.10215
	**398**	0.99999	0.00001	0.00000	0.10215		**800**	0.99992	0.00008	0.00000	0.10215
	**399**	0.99990	0.00010	0.00000	0.10215		**801**	0.00013	0.99892	0.00096	1
	**400**	0.99977	0.00023	0.00000	0.10215		**802**	0.99944	0.00056	0.00000	0.10215
	**401**	0.99998	0.00002	0.00000	0.10215		**803**	0.99995	0.00005	0.00000	0.10215
	**402**	0.99999	0.00001	0.00000	0.10215		**804**	0.99781	0.00219	0.00000	0.10215
