# Supplementary material for: ACE2 diversity in placental mammals reveals the evolutionary strategy of SARS-CoV-2
Source: Genet Mol Biol. 2020 Jun 8;43(2):e20200104. doi: 10.1590/1678-4685-GMB-2020-0104 (PMC7278419; doi:10.1590/1678-4685-GMB-2020-0104)
Supplement: Supplementary file 5 [file 1415-4757-GMB-43-2-e20200104-suppl5.pdf]

Supplementary Material to "ACE2 diversity in placental mammals reveals the evolutionary strategy of SARS-CoV-2"

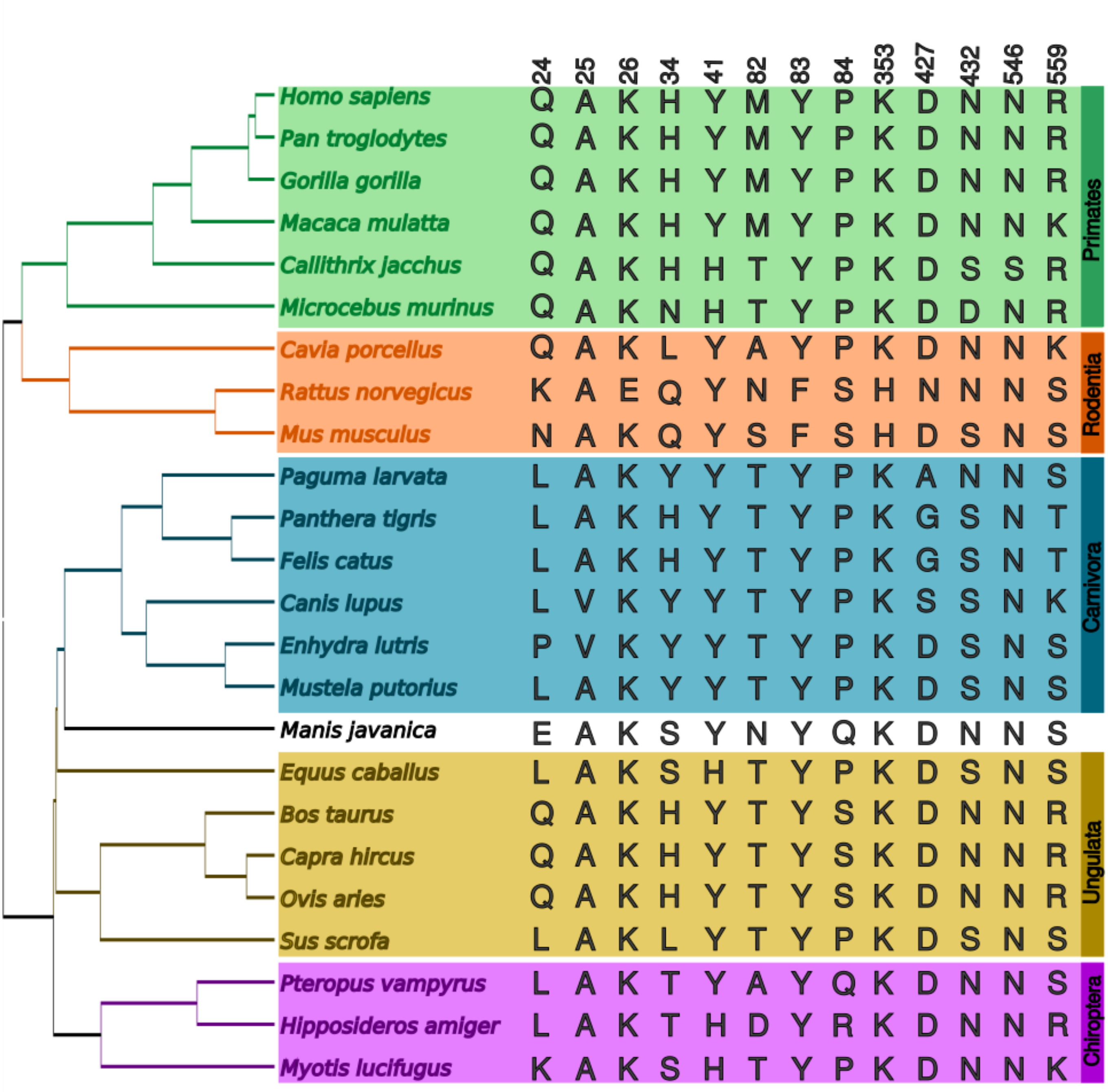

Figure S1 - Highlighted variable sites in placental mammalian ACE2 protein in the interaction with SARS-CoV-2.
